# The Role of Mutual-Help Groups in Extending the Framework of Treatment

**Published:** 2011

**Authors:** John F. Kelly, Julie D. Yeterian

**Keywords:** Alcohol use disorders (AUDs), alcohol dependence, treatment, treatment outcomes, recovery, self-help groups, mutual help groups (MHGs), Alcoholics Anonymous (AA), 12-step model, cost-effectiveness

## Abstract

Alcohol use disorders (AUDs) are highly prevalent in the United States and often are chronic conditions that require ongoing episodes of care over many years to achieve full sustained remission. Despite substantial scientific advances in specialized care, professional resources alone have not been able to cope with the immense burden of disease attributable to alcohol. Perhaps in tacit recognition of this, peer-run mutual-help groups (MHGs), such as Alcoholics Anonymous (AA), have emerged and proliferated in the past 75 years and continue to play an important role in recovery from AUDs. This article describes the nature and prevalence of MHGs, particularly AA, and reviews evidence for their effectiveness and cost-effectiveness and the mechanisms through which they may exert their effects. The article also provides details about how health care professionals can facilitate their alcohol-dependent patients’ participation in such groups and reviews the evidence for the benefits of doing so.

Alcohol-related disorders are highly prevalent in the United States, with an estimated 30 percent of Americans meeting diagnostic criteria for an alcohol use disorder (AUD) at some point in their life (Hasin et al. 2007). In addition, AUDs, especially alcohol dependence, often are chronic conditions that require numerous episodes of care over many years to achieve and maintain full remission (Dennis et al. 2005; White 2008). The professional health care system delivers a combination of pharmacological and behavioral interventions in an attempt to cope with the immense burden of disease attributable to alcohol (Room et al. 2005). However, professional resources have struggled to keep these problems in check by themselves. Perhaps in tacit recognition of this, peer-run mutual- help groups (MHGs), such as Alcoholics Anonymous (AA), have emerged and proliferated in the past 75 years (Kelly and Yeterian 2008*a*), and despite considerable advances in pharmacological and behavioral treatments for AUDs, these community groups continue to play an important role in helping millions of Americans achieve recovery. Indeed, MHGs are the most commonly sought source of help for alcohol and drug-use problems in the United States (Substance Abuse and Mental Health Services Administration [SAMHSA] 2008).

This article describes the role of MHGs, particularly AA, in the process of recovery from AUDs. It first describes the nature and prevalence of MHGs, reviews evidence of their effectiveness and cost-effectiveness, and examines the research on the mechanisms through which such groups may exert their effects. The article also explores how health care professionals can facilitate their alcohol-dependent patients’ participation in such groups and reviews evidence for the benefits of doing so.

## MHG Overview

MHGs—also known as self-help–groups—are groups of two or more people who share an experience or problem and who come together to provide problem-specific help and support to one another (Humphreys 2004). Members themselves run groups in rented venues, without professional involvement. And, unlike professional interventions, people can attend MHGs as intensively and for as long as they desire, without insurance approval or divulgence of personally identifying information. In contrast to professional treatments, people typically have access to MHGs at times when they are at higher risk of relapse, such as evenings and weekends, and many MHGs encourage members to contact each other by telephone between meetings whenever help is needed. Consequently, these organizations provide an adaptive, community-based system that is highly responsive to undulating relapse risk (Kelly and Yeterian 2008*b*).

AA is by far the most widespread MHG in the United States, with over 53,000 groups and 1.2 million members (AA 2008). Other substance-specific 12-step MHGs, such as Narcotics Anonymous, and Cocaine Anonymous, are numerous and can be found in most States. However, dual-diagnosis 12-step MHGs such as Double Trouble in Recovery (DTR), cognitive MHGs such as Women for Sobriety, and cognitive–behavioral MHGs such as SMART Recovery and Secular Organization for Sobriety are less common. In contrast to these groups, which all focus on abstinence from alcohol and drugs, one MHG, Moderation Management, focuses on limiting alcohol use to within safe limits and is designed to help the large number of nondependent “problem drinkers.” In addition to these more typical MHGs, the Internet has given rise to online meetings, which some people may find helpful either as an adjunct to, or instead of, face-to-face meeting attendance. People can find information on how to locate nearby face-to-face or online meetings on organizations’ Web sites or in the phonebook (for detailed listings and comparisons of MHGs, see Kelly and Yeterian 2008*a*).

### Evidence for the Effectiveness of MHGs

Because of its longevity, size, and influence, the vast majority of MHG research has focused on AA and other substance-focused 12-step groups, including some research on the dual-diagnosis 12-step group DTR (e.g., Magura 2008; Kelly and Yeterian 2008*b*). Researchers rarely perform randomized controlled trials on MHGs, in large part because participation in them is self-initiated, voluntary, and anonymous, making them difficult to randomize or control (see Kownacki and Shadish 1999 for an exception). That said, researchers still can obtain information on the effectiveness of MHGs through prospective, longitudinal studies that control for major confounders. Along these lines, it is important to note that researchers often draw study samples from treated populations rather than from community-based MHG populations, which may limit the generalizability of findings and make it difficult to estimate the independent contribution of MHGs to outcomes (Tonigan et al. 1996).

One long-term prospective study took this potential confounder into account by comparing outcomes in formally treated problem drinkers, informally treated problem drinkers (i.e., AA attendees), and untreated problem drinkers over a 16-year follow-up period (Moos and Moos 2006; Timko et al. 2000). At the 1- and 3-year follow-ups, half of the drinkers who self-selected into AA only were abstinent compared with about a quarter of those who self-selected into formal treatment. By the 8-year follow-up, 46 percent of those in formal treatment reached abstinence compared with 49 percent of the AA-only group. Drinkers in the study who self-selected into both AA and formal treatment also were more likely than those in formal treatment only to be abstinent at years 1 and 3 (42 percent and 51 percent vs. 21 percent and 26 percent) and, again, were not significantly different by year 8 (58 percent vs. 46 percent). Those who received formal treatment plus AA did not differ significantly from those in AA only across the follow-up in terms of abstinence rates (Timko et al. 2000). Additionally, a longer duration of AA attendance in the first 3 years independently predicted abstinence, as well as a lower likelihood of drinking problems at year 16 (Moos and Moos 2006). These findings indicate that for some people, MHG participation alone can serve as an effective intervention for AUDs.

Another naturalistic study of 3,018 male inpatients drawn from 15 Department of Veterans Affairs treatment programs found that after controlling for confounders, patients who attended only 12-step MHGs during a 1-year follow-up were more likely to be abstinent and free of alcohol-dependence symptoms than patients who received only outpatient treatment. Patients in the MHG-only group also were less likely to be depressed and had more friend resources after controlling for major confounders (Ouimette et al. 1998). However, patients attending both MHGs and outpatient treatment had the best outcomes on these variables. A further 2-year follow-up of 2,319 men from the same sample used structural equation modeling to examine the causal links between AA involvement and substance use (McKellar et al. 2003). Findings showed that AA involvement led to decreased alcohol consumption and fewer alcohol-related problems, after controlling for the level of patient motivation, comorbid psychopathology, and demographics. A mixed-gender community outpatient study, which used a rigorously controlled, lagged design to enhance causal inferences, similarly found that participation in MHGs led to subsequent improvement in alcohol-related outcomes (Kelly et al. 2006).

Questions sometimes arise as to whether MHGs are less suitable for certain populations. For example, because these groups focus purely on substance use and emphasize abstinence, some believe they may not appeal to people with dual diagnoses or people taking psychotropic or anti-relapse medications. In addition, it is believed by some that such groups may not resonate with atheists or agnostics because of their spiritual orientation, and that women and young people may not feel comfortable because they perceive MHGs to be male dominated and composed largely of middle-aged and older adults. Whereas the available empirical evidence suggests that these populations can benefit from participation in traditional AA or NA meetings, the benefits may be enhanced if people attend groups tailored more specifically to their individual needs, as with youth-oriented meetings (Kelly et al. 2005) and DTR, which is aimed at people with dual diagnoses (Kelly and Yeterian 2008*a*).

### Cost-Effectiveness of MHGs

Rising costs in health care make the question of cost-effectiveness increasingly important. Research suggests that involvement in MHGs may reduce the need for more costly professional treatment services. A 3-year prospective study of problem drinkers, for example, found that those who chose to attend only AA had overall treatment costs that were 45 percent lower than costs for people who chose to attend outpatient treatment, but outcomes were similar for both groups (Humphreys and Moos 1996). A large, multisite study of Veterans Affairs inpatient treatment programs that compared outcomes among substance-dependent patients who received either cognitive–behavioral treatment (CBT) or professional 12-step treatment found even bigger savings for MHGs ([Bibr b11-arh-33-4-350][Bibr b12-arh-33-4-350]). During a 1-year follow-up, patients in 12-step treatment programs participated in substantially more community AA and NA meetings, whereas patients in CBT programs utilized significantly more professional mental health services. As a result, annual costs for CBT patients were 64 percent higher than for 12-step patients, amounting to an additional $4,729 per patient. Notably, the patients in the two types of programs did not differ at intake on demographic and clinical characteristics, and their 1-year outcomes were similar, except that patients treated in 12-step programs had significantly higher rates of abstinence than those treated in CBT programs (46 percent compared with 36 percent; Humphreys and Moos 2001). In a subsequent 2-year follow-up of a matched sample, patients who received the 12-step treatment continued to show increased abstinence rates and higher levels of 12-step MHG participation. In addition, the CBT patients continued to rely more on professional services, resulting in 43 percent higher costs—an additional $2,440 per patient—during the second year post-treatment (Humphreys and Moos 2007).

### Mechanisms of Change in MHGs

Each MHG has its own implicit or explicit theory about how people achieve recovery. From AA’s perspective, people recover from alcohol dependence through a “spiritual awakening” or “psychic change” resulting from a combination of factors that include working the 12 steps, having a sponsor, believing in a “higher power,” and helping others (AA 2001). However, other theories also may explain how AA works. For example, the more implicit social component of AA meetings may promote therapeutic elements through group dynamics, such as the instillation of hope, vicarious learning and modeling, and altruism (Yalom 1995). In addition, empirical research on the mechanisms of change in AA highlights important cognitive, behavioral, and social factors associated with AUD remission (Kelly et al. 2009). For instance, several studies have found that the positive relationship between AA/MHG involvement and substance use outcomes can be explained by an increase in people’s social network and greater network support for abstinence (e.g., Humphreys and Noke 1997; Kaskutas et al. 2002; Kelly et al. 2010). Other studies have found that people who participate in AA have improved self-efficacy and motivation for abstinence, which in turn appears to mediate the relationship between AA participation and better outcomes (Connors et al. 2001; Morgenstern et al. 1997). In a study of DTR, Magura found that the relationship between DTR affiliation and abstinence was mediated by internal locus of control, which included internal motivation for change, coping skills, and self-efficacy (Magura 2008).

MHGs such as AA also appear to mobilize the same change processes—such as coping, motivation, and self-efficacy—that are mobilized by many different types of professionally led treatment (Kelly et al. 2009; Moos 2008). Hence, the positive effects of AA and other MHGs may not be tied to the specific technical content the groups contain. Rather, their chief strength may lie in their ability to provide free, long-term, easy access to recovery-related common therapeutic elements, the dose of which people can adaptively self-regulate according to their perceived need (Kelly et al. 2009).

In summary, evidence suggests MHGs such as AA can help people make and maintain beneficial changes to their alcohol use while also helping to reduce health care costs by providing a free and responsive recovery support system. These groups may help people recover by providing an ongoing recovery-specific social context that mobilizes active coping efforts, enhances self-efficacy, and continually remotivates people toward recovery. As such, MHGs appear to be a valuable resource that can serve as an important adjunct to, or, for some, an alternative to, professional care.

## Facilitating Participation in MHGs

Given that MHG participation appears to be an effective and cost-effective public health resource, it is important to consider how clinicians can best facilitate patient participation in such groups. These clinicians surely include those working at specialty substance-use-disorder facilities, but it is primary-care physicians, psychologists, psychiatrists, and emergency-room staff who typically first come into contact with people who meet the criteria for an AUD. Indeed, most people with an AUD do not seek treatment at a specialty substance-use-disorder facility (SAMHSA 2008) and, even when they do, they typically do not do so until 5 years after the onset of alcohol dependence (Wang et al. 2005). These statistics highlight how important it is that all types of health care providers routinely screen for AUDs and, when necessary, intervene (Kelly and McCrady 2008; National Institute on Alcohol Abuse and Alcoholism 2005).

One way to intervene is to facilitate patient participation in AA and other MHGs. To do that, clinicians—addiction specialists or not—can use strategies from 12-step facilitation (TSF) therapy (e.g., Project MATCH Research Group 1993). TSF is a professionally delivered intervention that is designed to educate patients about, and promote active engagement in, AA. For example, clinicians using TSF might help connect patients with current MHG members (Sisson and Mallams 1981; Timko et al. 2006) or help them prepare to attend an MHG meeting by dispelling myths and providing information about what to expect (Kaskutas et al. 2009; Walitzer et al. 2009). Clinicians also may monitor and discuss patients’ reactions to meetings and explore potential barriers to attendance (Donovan et al. 2003; Kelly and McCrady 2008). And they can deliver TSF effectively to groups of patients as well as individuals (Kaskutas et al. 2009).

When incorporating TSF strategies into practice, it is important for clinicians to keep an open mind about the utility of MHGs and have some degree of firsthand knowledge about groups such as AA. In fact, they may wish to attend local AA meetings that are open to the public and should become familiar with the times, locations, and various types of meetings available in their area, including meetings for beginners, for women only, or for young people. It also is useful for clinicians to develop a list of current and former patients who are willing to serve as AA contacts for new members.

There is evidence that primary-care providers can successfully incorporate TSF strategies into their practice. In the Medical Management treatment condition of the COMBINE study (Pettinati et al. 2004), providers focused on educating patients about addiction, providing them with support and optimism for recovery, and encouraging them to comply with medication regimens. They also described MHGs, such as AA, to patients as a helpful way to maintain sobriety and gave them MHG pamphlets along with phone numbers, times, and locations of meetings. The providers emphasized that MHG participation was voluntary but encouraged patients to try the groups even if they were reluctant or had had a negative experience with such groups in the past. Providers also recommended that patients try several different meetings to find a good match. Although data on the efficacy of this approach among non-specialty clinicians is not yet available, this study demonstrates the feasibility of implementing such approaches among addiction nonspecialists.

### Clinicians Can Make a Difference in Patients’ MHG Attendance

Several studies have demonstrated that clinicians can make a substantial difference in increasing the likelihood that patients will become and stay involved in MHGs. One early study found that when therapists had patients speak on the phone with current 12-step group members during an office visit and make arrangements to attend a specific meeting, every patient attended at least one meeting during the month following referral. In contrast, when therapists simply gave patients information about MHGs and encouraged them to attend a meeting, not one person attended (Sisson and Mallams 1981). In the large, randomized controlled trial on alcohol dependence called Project MATCH, participants in a condition that included TSF attended AA at a significantly higher rate during treatment and within the first 3 months of follow-up than those receiving CBT and motivational enhancement therapy (MET) (Tonigan et al. 2003). Another randomized controlled trial compared standard 12-step group referral, in which patients were given a schedule of local meetings and simply encouraged to attend, to intensive referral, which included several additional components, such as introducing patients to current AA/Narcotics Anonymous members and addressing patient concerns about attendance (Timko et al. 2006). At the 6-month follow-up, similar numbers of patients in both conditions attended a similar number of 12-step meetings. However, those in the intensive referral condition became significantly more involved in several aspects of the 12-step program; for example, they were more likely to have a sponsor and to report having had a spiritual awakening. These patients also improved more on alcohol and drug addiction severity scores than did patients in the standard referral condition.

### Evidence for the Beneficial Effects of TSF on Alcohol Use Outcomes

Along with improving MHG attendance, studies show that TSF also positively influences patients’ alcohol and drug-use outcomes. In Project MATCH, for example, TSF was as effective as the more empirically supported CBT and MET at reducing the quantity and frequency of alcohol use post-treatment and at 1- and 3-year follow-ups. Moreover, TSF was superior to CBT and MET at increasing rates of continuous abstinence, such that 24 percent of the outpatients in the TSF condition were continuously abstinent throughout the year after treatment, compared with 15 percent and 14 percent in CBT and MET, respectively (Tonigan et al. 2003). Abstinence rates at 3 years continued to favor TSF, with 36 percent reporting abstinence, compared with 24 percent in CBT and 27 percent in MET (Cooney et al. 2003).

Another study examined the incremental effects of incorporating TSF into an empirically supported cognitive–behavioral intervention, called Social Skills Training (SST). The study compared SST alone with two other therapies that combined SST with TSF, but delivered them using two different therapeutic styles; one was more directive, spending 38 percent of session time discussing AA participation and encouraging AA attendance; the other was more client centered, based on the principles of motivational interviewing (Walitzer et al. 2009). Patients in the directive TSF condition attended more AA meetings, became more involved in the AA program, and had a higher percentage of days abstinent than those receiving SST alone or the motivational interviewing–based TSF approach. Because the TSF motivational condition spent only 20 percent of session time discussing AA and SST alone spent only 8 percent of session time discussing AA, it suggests that more time spent focusing on AA and/or being more directive about attending AA meetings may be optimal for enhancing AA participation and improving alcohol use outcomes.

In yet another randomized study of alcohol-dependent outpatients (Litt et al. 2009), researchers attempted to increase social network support (NS) for abstinence by systematically encouraging patients to exploit the social aspects of AA. They compared this intervention with two other cognitive-behavioral treatment interventions and found that study participants in the 12-step NS group were abstinent 20 percent more days than participants in the other conditions and were more involved in AA at 2-year follow-up. Furthermore, AA participation and the number of abstinent friends in the NS condition partially mediated this treatment effect.

## Conclusions

More than 10 years ago, a provocative article (Humphreys 1997) pointed out the dearth of research and clinical attention afforded to community mutual-help resources for alcohol-related problems. Since then, a large number of high-quality studies have emerged highlighting the utility of MHGs and the efficacy of professional interventions designed to facilitate their use. Although most research has focused on AA, other MHGs may hold similar utility but need further study. AA appears to be helpful to a broad array of people and is highly cost effective. In addition, clinical facilitation of patients’ participation in MHGs may lower health care costs by reducing reliance on professional resources and also is likely to enhance patients’ outcomes. As the treatment field moves toward recovery-oriented systems of care (Kelly and White 2011), continuing to forge stronger links between community-based and professional treatment resources will allow for a more efficient systemic approach to alleviating the suffering and prodigious social costs associated with AUDs.
